# Supplementary material for: Recombination junctions from antibody isotype switching classify immune and DNA repair dysfunction
Source: Nat Commun. 2025 Dec 19;16:11331. doi: 10.1038/s41467-025-67206-5 (PMC12722312; doi:10.1038/s41467-025-67206-5)
Supplement: Supplementary file 1 — Supplementary Information [file 41467_2025_67206_MOESM1_ESM.pdf]

Supplementary Information

“Recombination junctions from antibody isotype switching classify immune and DNA repair dysfunction”

Vázquez García C.*, Obermayer B.*, *et al.,*

*corresponding author: kathrin.delarosa@helmholtz-hzi.de

Page Content

Page	Content
02	Supplementary Figure 1: SWIBRID pipeline and benchmarking of CSR junction PCRs
04	Supplementary Figure 2: CSR junction characteristics
06	Supplementary Figure 3: SWIBRID re-analysis of CSR junction short read data generated via the HTGTS CSR assay from Panchakshari et al.
08	Supplementary Figure 4: SWIBRID stratifies CVID patients
10	Supplementary Figure 5: Gating strategy for IgA+IgG+ quantification in Supplementary Fig. 4e
11	Supplementary Figure 6: Gating strategy of donor A, B, C in Figure 1f,g
12	Supplementary Figure 7: Uncropped gel from Supplementary Figure 1b
12	Supplementary Figure 8: Uncropped gel from Supplementary Figure 1j
13	Supplementary Figure 9: Histogram of gap sizes in the MSA of different samples
13	Supplementary Figure 10: Determining a dendrogram cutoff
13	Supplementary Figure 11: Cluster filtering strategy
14	Supplementary Figure 12: Cluster vs. clone number in synthetic data
14	Supplementary Figure 13: Comparing exact and approximate linkages
15	Supplementary Note 1: Rationale for clustering strategy
18	Supplementary Note 2: Estimating reproducibility by cluster tracing
25	Supplementary Table 1: Primers used in CSR joint PCR
26	Supplementary Table 2: Primers used in BCR transcript sequencing
27	References

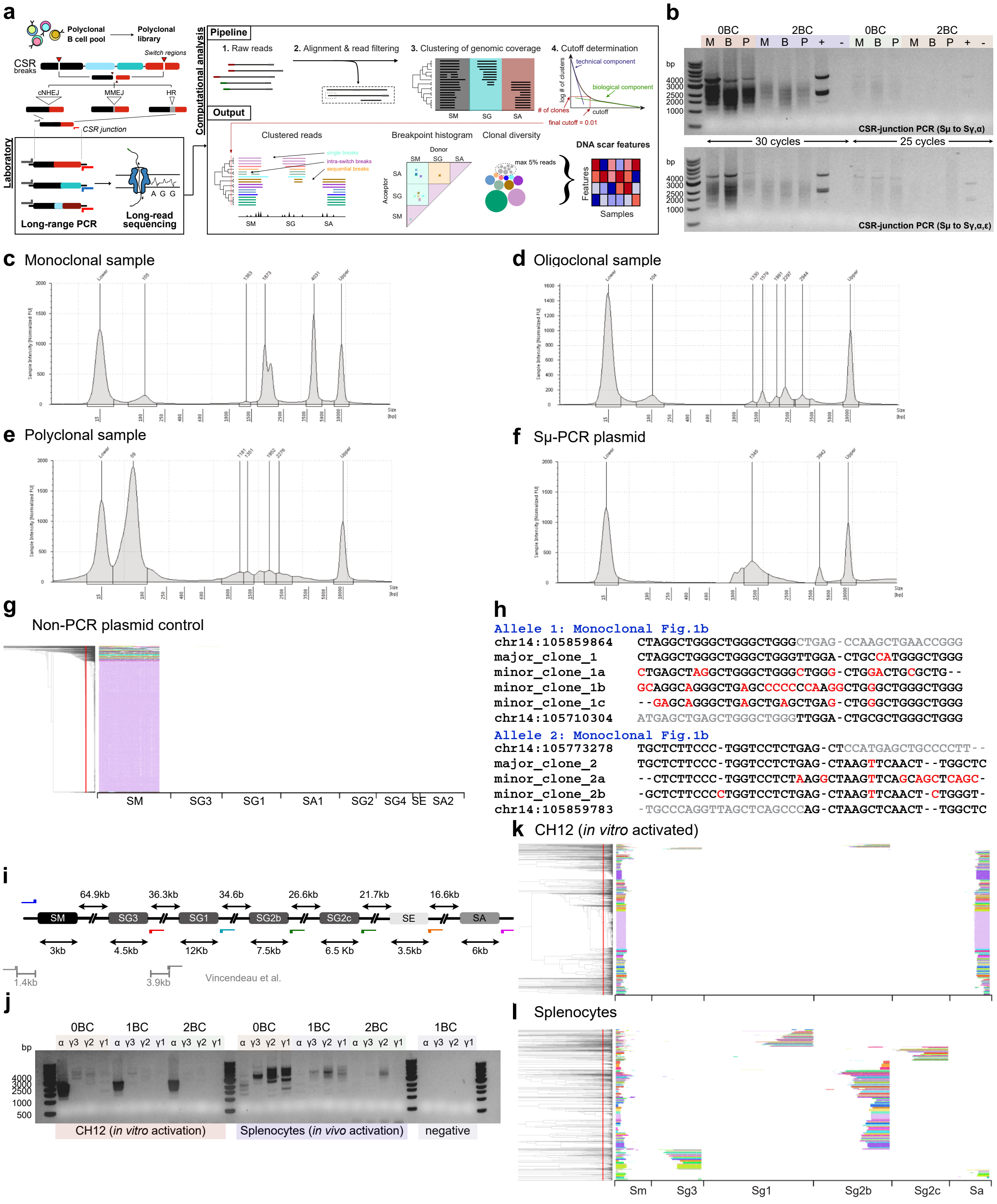

Supplementary Figure 1. SWIBRID pipeline and benchmarking of CSR junction PCRs. (Figure legend on the next page).

Supplementary Figure 1. SWIBRID pipeline and benchmarking of CSR junction PCRs. a. Scheme of library preparation and data analysis workflow. **b.** Representative human CSR junction PCRs using indicated materials were analyzed by agarose gel electrophoresis. The smear reflects the size distribution of CSR junctions from polyclonal samples. Single bands represent the amplification of defined junctions from monoclonal cell lines. Left: 30 PCR cycles; Right: 25 PCR cycles. Top: CSR junction PCR with Sμ forward, Sγ, and Sα reverse primers. Bottom: Sμ forward, Sγ, Sα, and Sε reverse primers. M = 25,000 $CD27^+$ IgG^+ IgA^+ B cells, B = 50,000 $CD19^+$ PBMCs, P = 200,000 PBMCs, “+” = positive control deriving from a monoclonal EBV-immortalized cell line with alleles switched to Sγ and Sα, respectively. “-“ = negative control without template. **c.** TapeStation results of PCR amplicons obtained from CSR junction of samples from Fig.1b and Supplementary Fig.1e, namely, the monoclonal B cell line, **d.** an oligoclonal, EBV-immortalized B cell pool, e. 25,000 IgG/A^+ human B cells, and **f**, a plasmid containing the human Sμ region was linearized via a restriction enzyme digest, representing an outcome in absence of PCR amplification. **g.** Read plot obtained from the linearized plasmid with 2,500 reads. **h.** Multiple sequence alignments of representative reads from major and minor clones associated with the two alleles of the monoclonal cell line (Fig. 1b) across the SM-SA1 and SM-SG3 junctions, respectively. Switch region reference sequence (hg38) is indicated in gray. Mismatch nucleotides are highlighted in red. **i.** Scheme of the mouse *Igh* locus, with forward and reverse primer positions indicated. Primers used by Vincendeau et al.[30] are depicted in light grey. **j.** Agarose gel electrophoresis of PCR amplicons obtained from mouse CSR junctions. Left: *in vitro* activated CH12 cells. Right: *ex vivo* mouse splenocytes. The Sμ forward primer was combined with either Sγ1, Sγ2, Sγ3 or Sα as indicated. 0BC = primers without barcodes were used, 1BC = the forward primer contained a barcode, 2BC = forward and reverse primers contained barcodes. **k.** Read plot of a CH12 sample and splenocytes.

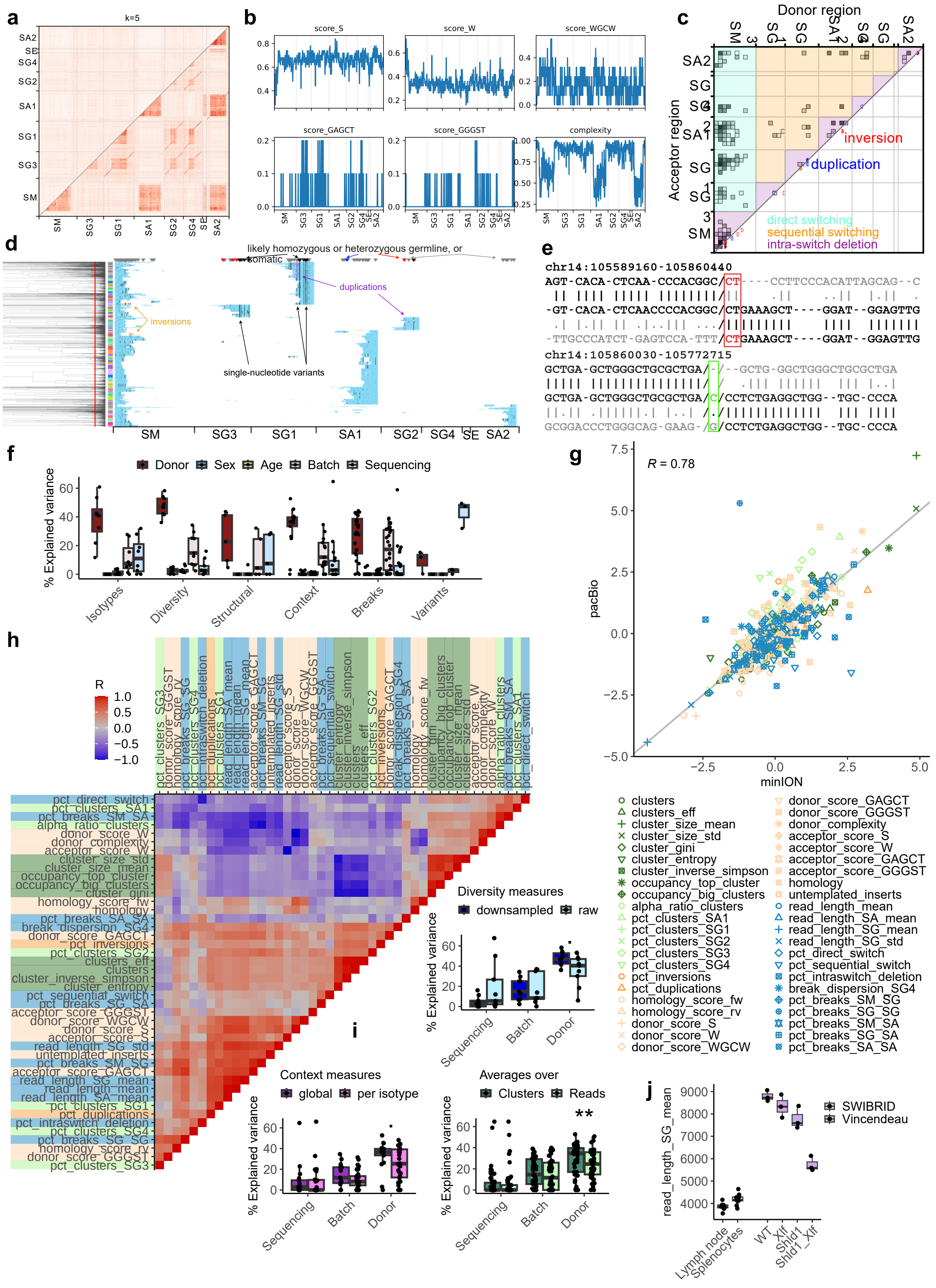

Supplementary Figure 2. CSR junction characteristics. (Figure legend on the next page).

Supplementary Figure 2. CSR junction characteristics. **a.** Depiction of 5mer homology in 50nt bins between different human switch regions. Forward homology is shown in the lower right diagonal, reverse-complementary homology in the upper left diagonal. The features homology_score_fw and homology_score_rv are derived by weighting this homology score with the frequency of breakpoints. **b.** Motif frequency scores and sequence complexity used to create associated features by weighting with breakpoint frequencies along the human IGH locus in 50nt bins, S=G/C, W=A/T. **c.** Classification of break type and depiction of inversions and duplications in a two-dimensional plot visualizing class-switch recombination events (see also Fig. 2a). **d.** Illustration of features related to structural aberrations or single-nucleotide variants in the read plot of the polyclonal B cell pool of Fig. 1b; inversion events are colored in orange, duplication events in violet. **e.** Illustration of sequence context features homology (top) or untemplated_inserts (bottom) for two exemplary reads from WT CH12 cells. Read sequence (middle) is re-aligned to genomic sequence surrounding the upstream (top) and downstream (bottom) breakpoint. Alignment block boundaries are indicated by the slashes. **f.** Box plots of percent explained variance by donor, sex, age, batch, or sequencing method, for n=70 features in indicated categories. **g.** Scatter plot of feature values (as z-scores) for samples from 10 donors that were sequenced with PacBio or Nanopore technology. Overall correlation (R) is indicated. **h**. Heatmap of feature-to-feature correlation values using the C2 cohort. Background colors on axis as in Fig. 2a. **i.** Comparison of explained variance by donor, batch, or sequencing methods for diversity measures calculated using all reads or on samples downsampled to 1,000 reads (top); for context measures calculated globally or per isotype (lower left); for context and break features calculated by averaging over reads or clusters (lower right). P-values from two-sided Wilcoxon test. * $p < .05$. **j.** Mean fragment length of reads in different samples from mouse lymph nodes, splenocytes, or from data of Vincendeau et al.[30], where a different primer design leads to much longer PCR products (see Supplementary Fig. 1i). Boxes in f,i and j indicate 25th to 75th percentile; whiskers extend to largest/smallest value no further than 1.5x interquartile range, lines indicate median.

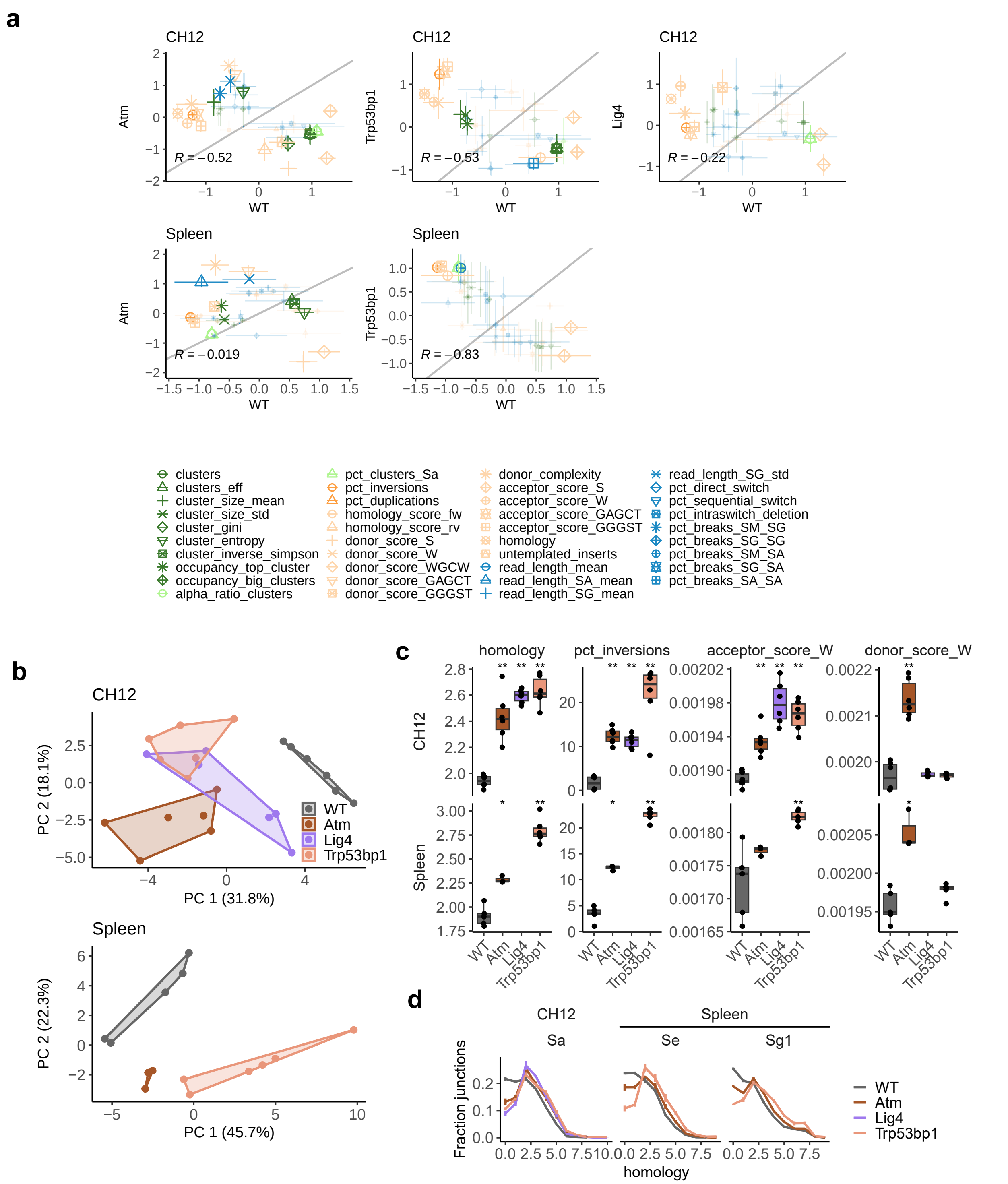

Supplementary Figure 3. SWIBRID re-analysis of CSR junction short read data generated via the HTGTS CSR assay from Panchakshari et al.[27] (Figure legend on the next page).

Supplementary Figure 3. SWIBRID re-analysis of CSR junction short read data generated via the HTGTS CSR assay from Panchakshari et al.[27] **a.** Comparison of scaled mean SWIBRID features of different genotypes in CH12 and splenocytes. Significantly different features are indicated using larger symbols (adj. p-value < 0.05 from two-sided t-test compared to WT (n=3-6 replicates per group) with Benjamini-Hochberg correction). **b.** PCA of re-analysed CH12 (top) and splenocyte (bottom) using 39 robust features. **c.** Box plots of selected SWIBRID features. It should be noted that spleen data lack Lig4 KO samples (**: p < 0.01 from two-sided Wilcoxon test). **d.** Analysis of homology used for repair. Error bars indicate s.e.m.

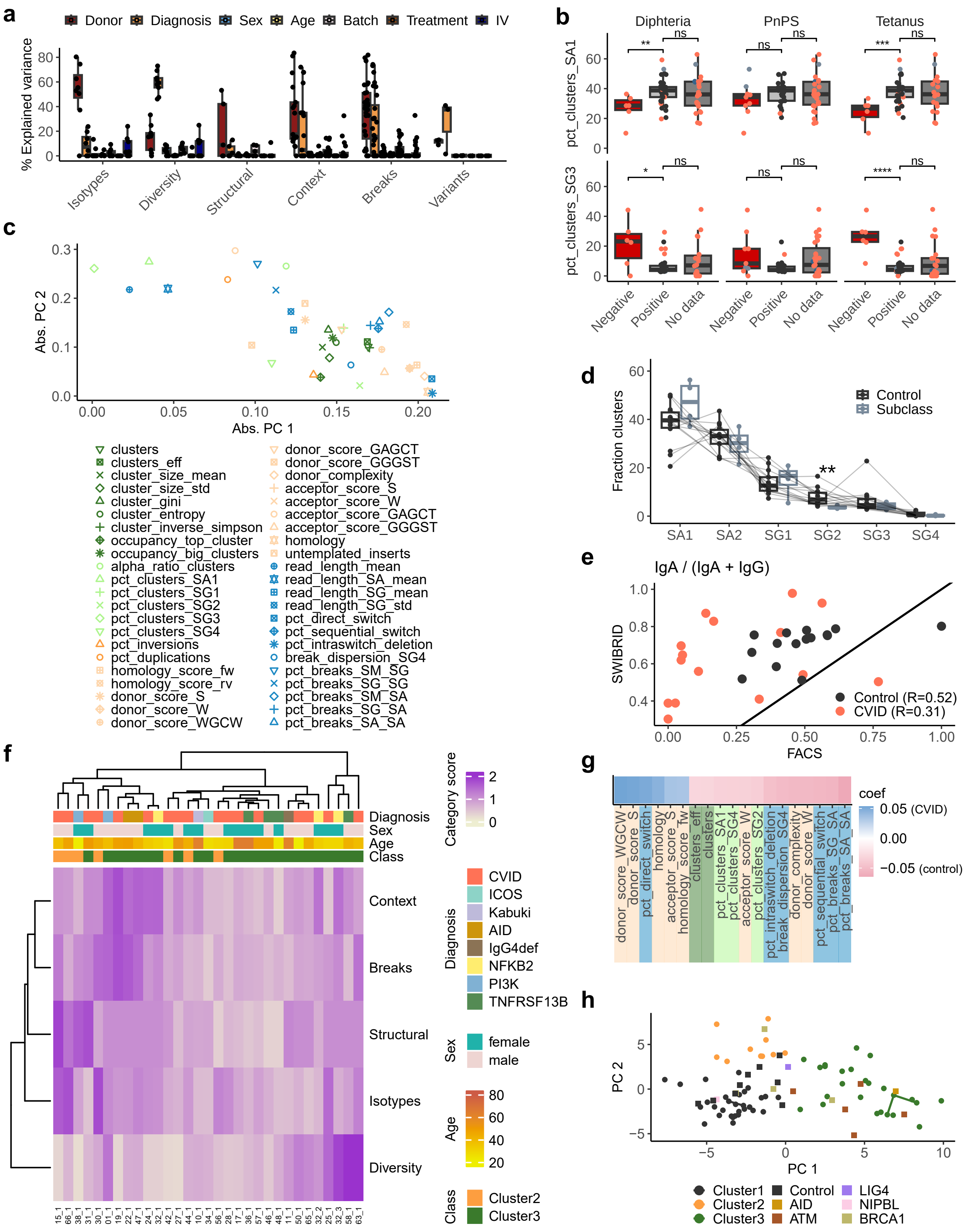

Supplementary Figure 4. SWIBRID stratifies CVID patients. (Figure legend on the next page).

Supplementary Figure 4. SWIBRID stratifies CVID patients. a. Box plots of percent explained variance by donor, diagnosis, sex, age, batch, treatment, or Ig administration for n=70 features in indicated categories. **b.** Box plots of indicated feature values for groups that test positive or negative for serum IgG against diphtheria, pneumococcal (PnPs), or tetanus antigens. No data: antibody titers were not measured. **c.** (Absolute) feature loadings for the PCA of Fig. 4e. **d.** Fraction of clusters with different isotypes for donors with subclass deficiencies or controls. **e.** Scatterplot comparing relative proportions of IgA B cells from a FACS analysis against Sα junctions detected in SWIBRID. Pearson correlation values indicated for CVID patients or controls, respectively. **f.** Heatmap of category scores (sum of absolute z-scores of all features in a category) for CVID and CVID-like samples. **g.** Top 19 coefficients in a multinomial ridge regression model derived from the 29 training samples. Features are colored according to the classification of Fig. 2a. Higher coefficients indicate higher values in the CVID group. **h.** PCA of Fig. 4d,e colored by class membership from Fig. 4h, together with data points from DNA repair set (Fig. 4f). P-values in b and d from two-sided Wilcoxon test (****: $p < 0.0001$, ***: $p < 0.001$, **: $p < 0.01$, *: $p < 0.05$). Boxes in a,b and d indicate 25th to 75th percentile; whiskers extend to largest/smallest value no further than 1.5x interquartile range, lines indicate median.

A

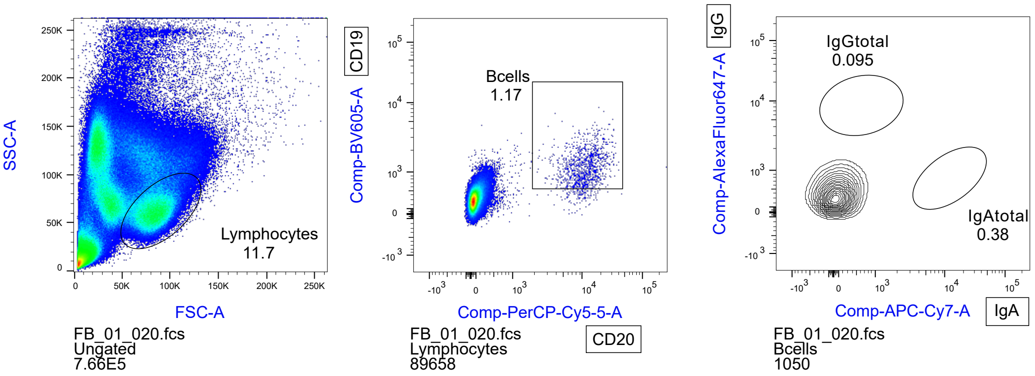

B

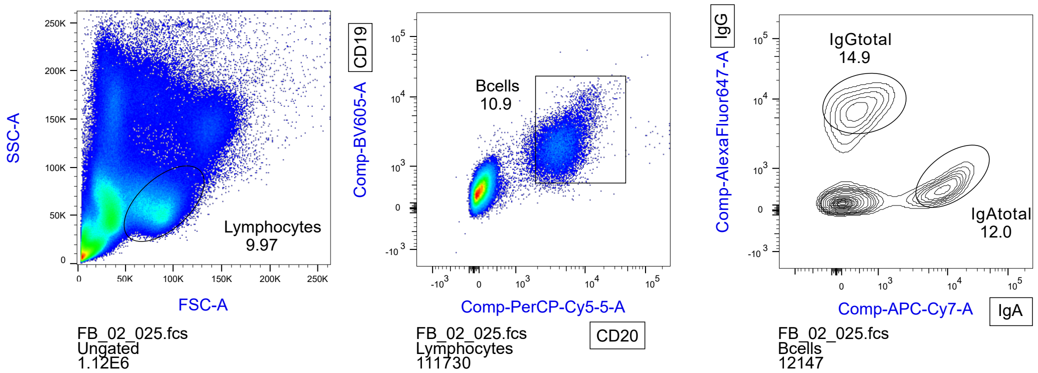

Supplementary Figure 5. Gating strategy for IgA+, IgG+ quantification in Supplementary Fig. 4e. A. Example of immunodeficient donor FB_01 (Hyper-IgM, PI3K-deficient). **B.** Example of a healthy donor FB_02. Quantification of IgG and IgA B cells for comparison with SWIBRID results was done using the antibodies: IgG-AF647 (dilution: 1:500, Dianova, #109-606-170); IgA-APC-Vio770 (dilution: 1:170, Miltenyi Biotec, #130-113-473, clone IS11-8); CD19-Brilliant Violet 6005 (dilution: 1.160, Becton Dickinson, #562653) and CD20-PerCP-Vio700 (dilution: 1:170, Miltenyi Biotec, #130-113-377, clone LT20) in a BD LSRFortessa™ Cell Analyzer.

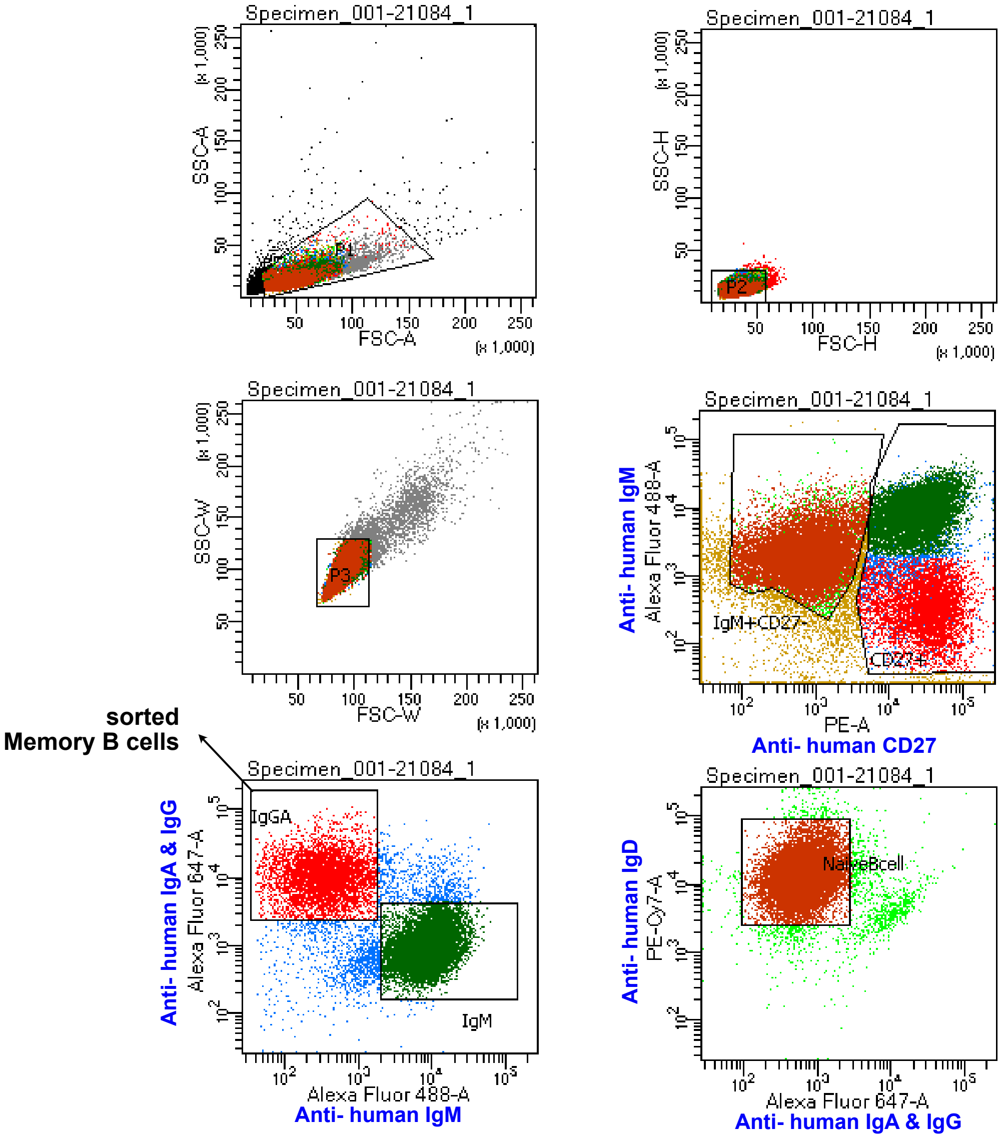

Tube: 21084_1

Population	#Events	%Parent	%Total
All Events	50,000	####	100.0
P1	46,267	92.5	92.5
P2	46,093	99.6	92.2
P3	43,700	94.8	87.4
IgM+CD27-	15,944	36.5	31.9
NaiveBcell	14,597	91.6	29.2
CD27+	18,531	42.4	37.1
IgGA	5,445	29.4	10.9
IgM	11,211	60.5	22.4

Supplementary Figure 6: FACS Gating Strategy for memory B cells in donor A, B, C in Figure 1f,g. PDF created from the BD FACSDiva™ Software during the sorting of donor A. Same gating strategy was used for donor B and C. Sorted memory B cells are highlighted. Sorting was done using CD27-PE (dilution: 1:170, Miltenyi Biotec, #130-114-156, clone M-T271); IgD-PE-Cy7(dilution: 1:80, Miltenyi Biotec, #130-098-584), IgM-AF488(dilution: 1:1000, Life Technologies, #A21215); IgG-AF647(dilution: 1:500, Dianova, #109-606-170); IgA-AF647 (dilution: 1:500, Dianova, #109-606-01) in a BD FACSAria™ Fusion Flow Cytometer.

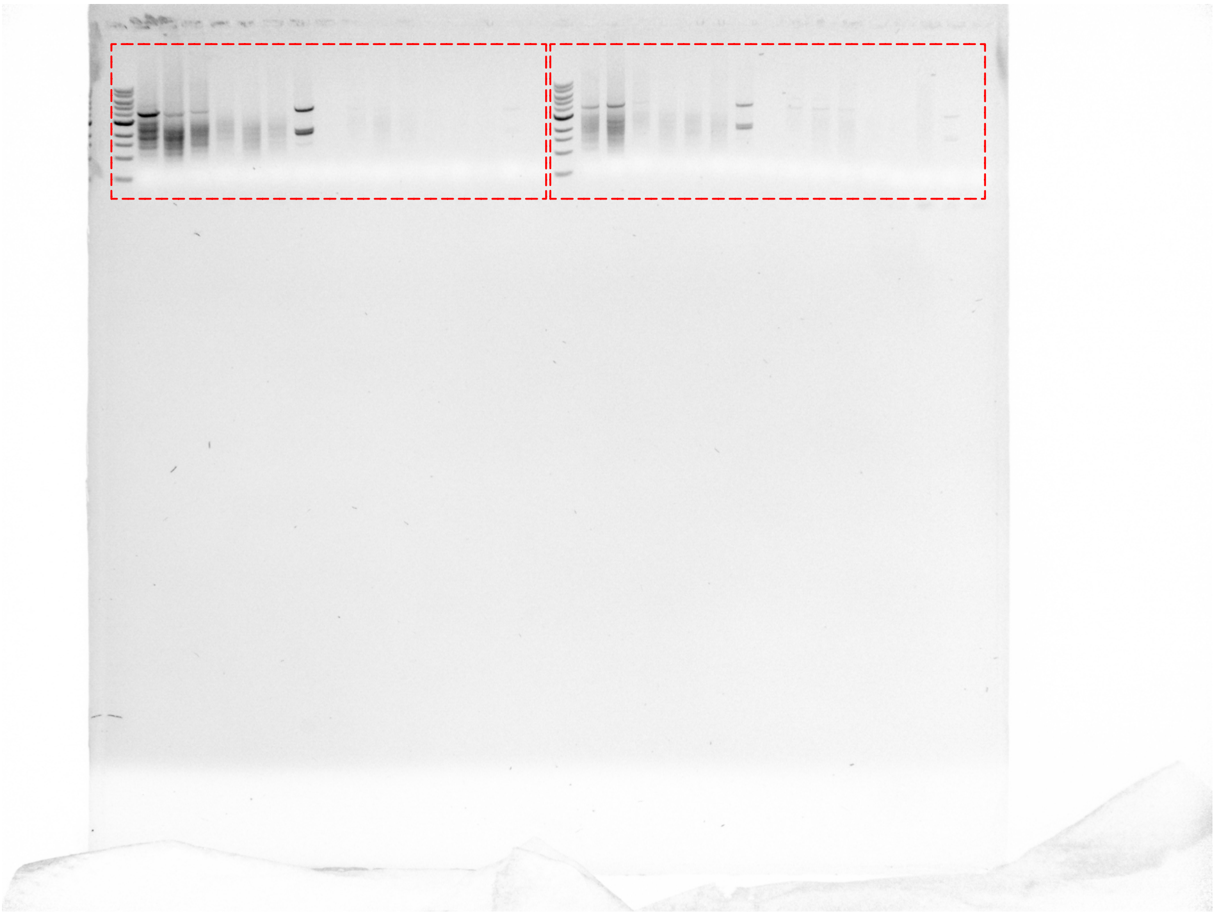

Supplementary Figure 7: Uncropped gel - Supplementary Figure 1b. Cropped image is highlighted in red.

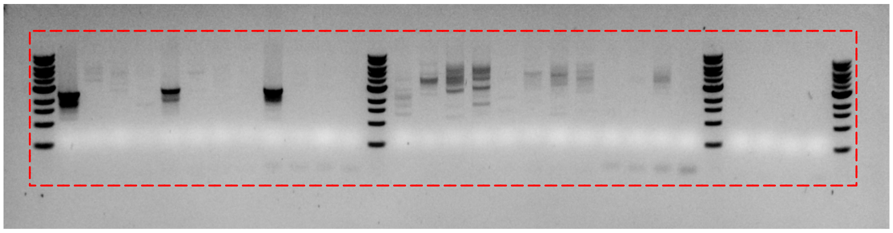

Supplementary Figure 8. Uncropped gel - Supplementary Figure 1j. Cropped image is highlighted in red.

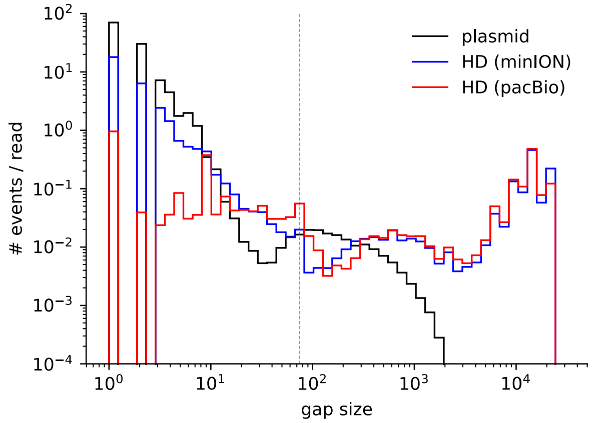

Supplementary Figure 9. Histogram of gap sizes in the MSA of different samples. A non-PCR, linearized plasmid control and pooled data from 10 healthy donors sequenced with either minION or pacBio technology. Dashed vertical line indicates the 75nt cutoff separating likely artefactual from presumably real breaks.

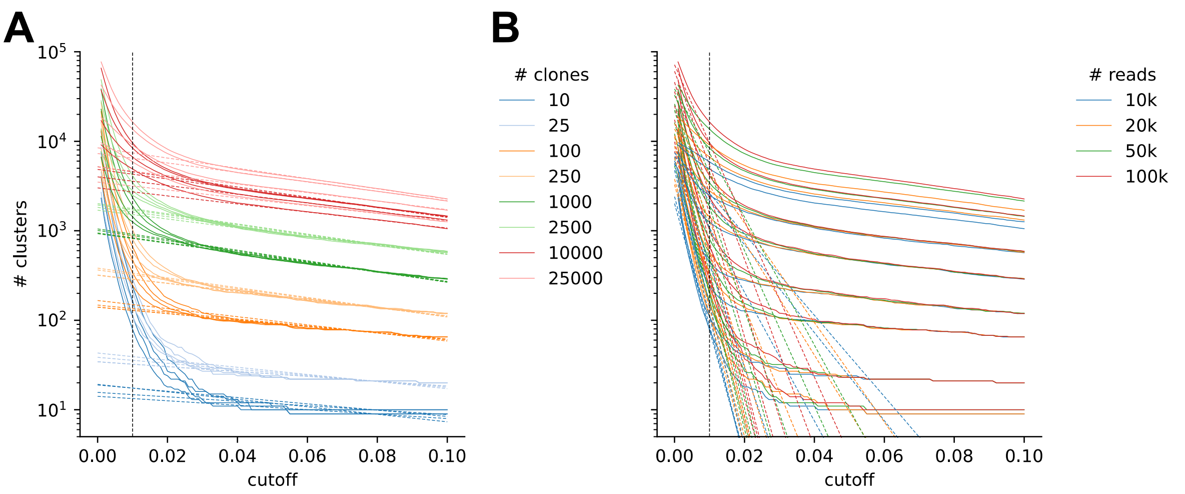

Supplementary Figure 10. Determining a dendrogram cutoff. Number of clusters as a function of dendrogram cutoff, colored by number of input clones (A) or number of input reads (B), together with one component of a double-exponential fit tailored to capture the trends at high or low cutoff respectively. Vertical line indicates cutoff value of 0.01.

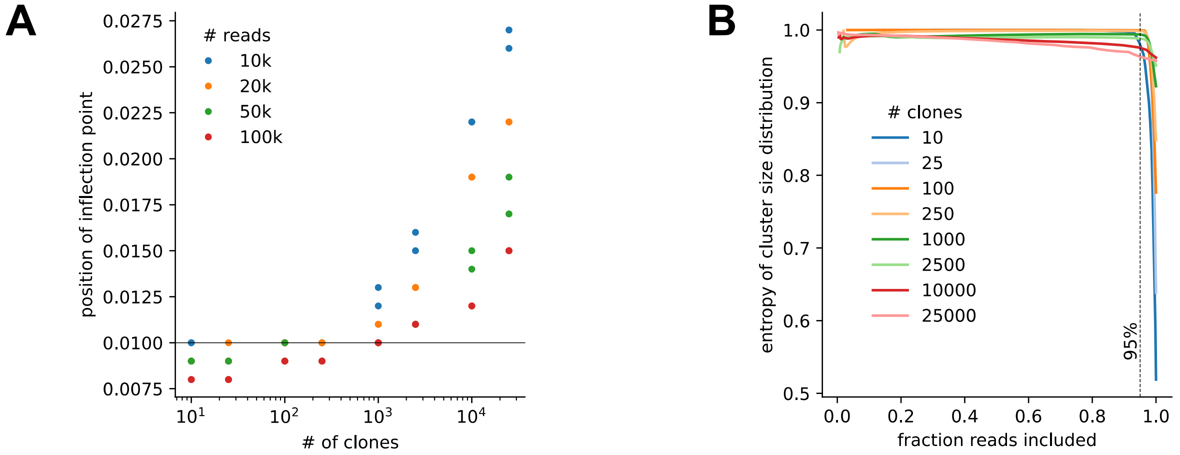

Supplementary Figure 11. Cluster filtering strategy. Fixed cutoff as a function of synthetic and real data **A**. position of inflection point in the curves of Supplementary Figure 10 for different input numbers of reads and clones. Horizontal line indicates cutoff value of 0.01. **B**. Entropy of cluster size distribution as function of the fraction of reads used when including bigger clusters first (for 50k input reads). Dashed vertical line indicates 95% cutoff.

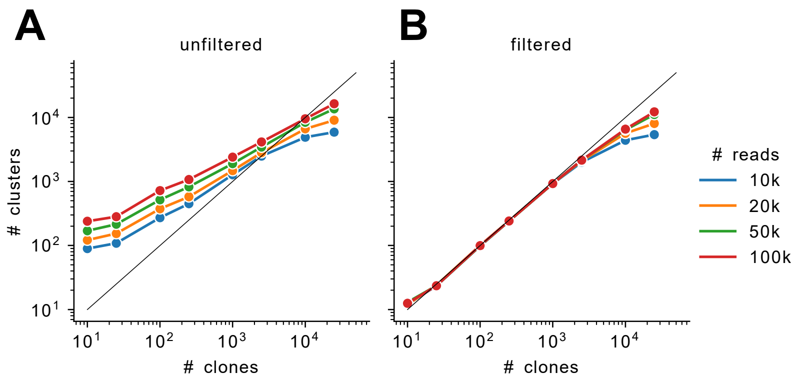

Supplementary Figure 12. Cluster vs. clone number in synthetic data. Number of clusters as a function of input clone number, for different numbers of input reads. **A.** unfiltered cluster number, **B.** cluster number when considering only the biggest clusters containing at least 95% of reads.

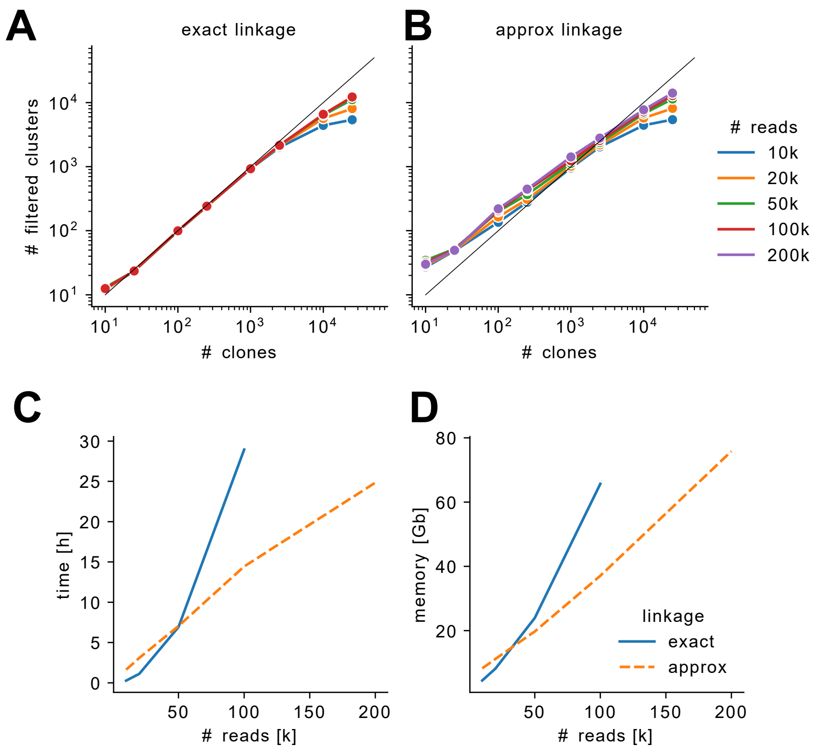

Supplementary Figure 13. Comparing exact and approximate linkages. Comparison of an exact linkage created by fastcluster (A) with an approximate linkage generated using sparsecluster (B). Time (C) and memory (D) requirements for exact and approximate linkage generation.

Supplementary Note 1: rationale for clustering strategy

Clustering on the order of tens of thousands of reads of 2-5 kb length presents unique challenges. In this case, these include the lack of information on the true cluster number *a priori*, even by order of magnitude, the size of true differences between different clones, and the likely substantial amount of technical noise due to sequencing and mapping.

We decided to use hierarchical clustering to take advantage of i) the naturally induced one-dimensional ordering by the resulting dendrogram, which we used for read plots, and ii) its structure that optimally resolved the presumably hierarchical structure of the data: different isotypes present large coarse clusters that are subdivided into smaller clusters, potentially even with hierarchical substructure due to different clones sharing donor or acceptor break points. We further opted to avoid clustering read sequences themselves via a true multiple sequence alignment, which would be very resource intensive and suffer from technical noise. Instead, we transformed (genome-)aligned read sequences into a pseudo multiple sequence alignment, encoded as a sparse matrix of dimensions #reads by #positions, which can be clustered easily and much more efficiently using low-level math routines. Restricting the *Igh* locus to relevant intronic regions leaves us with about 30k positions, of which roughly 10% are used for any given read. Given a high rate of sequencing errors, only the coverage of a position (not nucleotide identity) was considered. Further, long-read sequencing is prone to create indels. Insertions are ignored in the pseudo multiple sequence alignment, but are taken into account at other points in the pipeline if they are either templated and detected by mapping, or untemplated and occurring around larger break points, in which case they are identified in the breakpoint realignment step. We noticed that deletions of up to 75nt length appear frequently, even in a non-PCR plasmid control, but much less often when using pacBio instead of minION sequencing technology (Supplementary Figure 9). To increase the robustness of the clustering by preferentially comparing the positions of larger breaks rather than likely random small deletions, we ignored all gaps smaller than 75nt. Identification of structural rearrangements (duplications and inversions) was enabled by encoding coverage as integer values, where 1x means regular 1-fold coverage, 2x signifies a duplication event, and -1x an inversion. In that encoding, two reads (=rows of the matrix) can be quickly compared using cosine distance, and hierarchical clustering can be performed using a very fast implementation in the fastcluster python package.

Average (UPGMA) linkage demonstrated optimal performance based on visual inspection of resulting breakpoint positions per cluster and the presence of cluster-specific (likely somatic) single nucleotide variants. We next devised a strategy for choosing the dendrogram cutoff and designed a post-clustering filter.

To address these issues, we performed extensive simulation studies using the test mode of the SWIBRID pipeline, taking as input SWIBRID results for a pool of human donors in the form of alignment block coordinates (usually 2 blocks for one switch event; bed format). The simulation creates reads from the associated genomic sequence, and adds mutations, insertions, and deletions according to parameters estimated on the non-PCR plasmid control. Simulations were done for a number of clones ranging from 10 to 25,000, and for a total number of reads between 10,000 and 200,000.

Plotting the resulting number of clusters (on a log-scale) as function of the dendrogram cutoff resulted in curves exhibiting a two-exponential decay pattern, with slopes (roughly) independent of number of input reads or clones, and the intercepts (prefactors) strongly correlated to input read or clone number (Supplementary Figure 10).

This behavior suggested that the trend at low cutoff values is mainly driven by technical noise (rapidly increasing the number of clusters), while the trends at high cutoff values reflect true biological variability. A cutoff value to suppress the noise while keeping as much of the biological variability as possible would therefore lie near the intersection of the two trends, or near the inflection point of the curve. On the synthetic data, these inflection points show a residual dependence on input read and clone number, which is minimal around a cutoff value of 0.01 (Supplementary Figure 11A). Fitting these trends on actual data yielded less robust and somewhat unstable estimates. We therefore decided to adopt this fixed cutoff value to ensure that different runs remain comparable.

We further noticed small isolated clusters in our synthetic data that were likely the result of technical noise, while “true” clones had a relatively even size distribution (specifically, the Poisson model was used). This suggested that we could filter out spurious clusters by omitting reads from the smallest cluster. Plotting the entropy of the cluster size distribution as function of the fraction of reads used (bigger clusters first), elicited a sharp drop in the entropy around 95% of reads included, in line with the idea that spurious small clusters decrease the entropy (Supplementary Figure 11B).

Adopting this additional 95% cutoff in the end leads to an agreement between input clone number and resulting filtered cluster number, largely independent of the input read number over several orders of magnitude (Supplementary Figure 12). The unfiltered cluster number strongly overestimates the clonality, especially for samples with low clonality, and shows a substantial dependence on the number of input reads. A saturation effect was observed when the input clone number approaches the input read number.

Typical read numbers per sample in our experiments showed a relatively wide distribution from below 1,000 to several 100,000 reads. Although results in the simulation experiments were largely independent of input read number, we wanted to ensure that samples with different read numbers are comparable. Larger read numbers also lead to performance issues in terms of required CPU time and memory. Therefore, the read number used for clustering was capped at 50,000. Nevertheless, to make our method scalable, we implemented another clustering strategy based on the construction of an approximate nearest-neighbor graph followed by graph-based hierarchical clustering[1] . This method, implemented in a novel python package sparsecluster, allows for sparse input, and exhibits superior time and memory efficiency, while showing similar performance (Supplementary Figure 13).

Supplementary Note 2: estimating reproducibility by cluster tracing

In order to estimate how reproducibly we can detect and characterize individual switching events in different samples, we devised a meta-clustering strategy to identify recurring clusters. For this, we averaged the pseudo multiple sequence alignments across all reads per cluster in a given sample and created a meta-alignment by pooling these averaged clusters from different samples. This meta-alignment was clustered using the same hierarchical clustering approach (cosine metric, average linkage) and cluster cutoff as before. Clusters from different samples assigned to the same meta-cluster are considered recurring events. Below, we show top recurring events between different samples together with read alignment to genomic regions upstream and downstream of CSR junctions or templated inserts. While the exact coordinates of the junctions and the number of untemplated or homologous nucleotides surrounding them do not always agree exactly, many of these events (templated insertions or intra-switch deletions) are sufficiently characteristic to make accidental agreements highly unlikely.

Example 1 (page 20, 21, 22 – Supplementary Data 1) shows SWIBRID read plots for 3 independent replicates of 50000 class-switched memory B cells from the same donor. The top 15 shared clusters are highlighted with matching colors across the three plots, and recurring templated insertions are indicated in black. E.g., one templated insertion (#16; chr3:141391308-141391553) occurs within SM (between chr14:105860269 and chr14:105860271) followed by a break from chr14:105860076 to SG1 (chr14:105744764) that is associated with around 40 untemplated nucleotides. Another templated insertion (#17; chr16:22336035-22336493) occurs within SA2 (between chr14:105589576 and chr14:105589587), with the main switch junction from SM (chr14:105860866) to SA2 (chr14:105589849) again featuring around 50 untemplated nucleotides. Recurring intra-switch deletions are repeatedly observed within SM, once marked as #13 in dark red at chr14:105859194-105860296 followed by a break to SA1 (from chr14:105859001 to chr14:105710805), with several homologous nucleotides around both breaks, and once marked as #12 in yellow at chr14:105860418-105859294 followed by a break to SA2 (from chr14:105858630 to 105589508).

Example 2 (page 23, 24 - Supplementary Data 1) shows read plots for a pool of amplicons generated from PBMCs of a healthy donor that were subsequently sequenced with

minION or pacBio technology, respectively. In this case, the largest clusters are clearly matched in relative size, and easily recognizable events include a sequential switching event marked as #3 in blue from SM (chr14:105860419) to SA1 (chr14:105709995-105710378) to SA2 (chr14:105589053), or a simple junction without untemplated or homologous nucleotides marked as #13 in dark red from SM (chr14:105860677) to SA1 (chr14:105709826).

top recurring clusters in replicate 1 of 50000 B cells of donor A

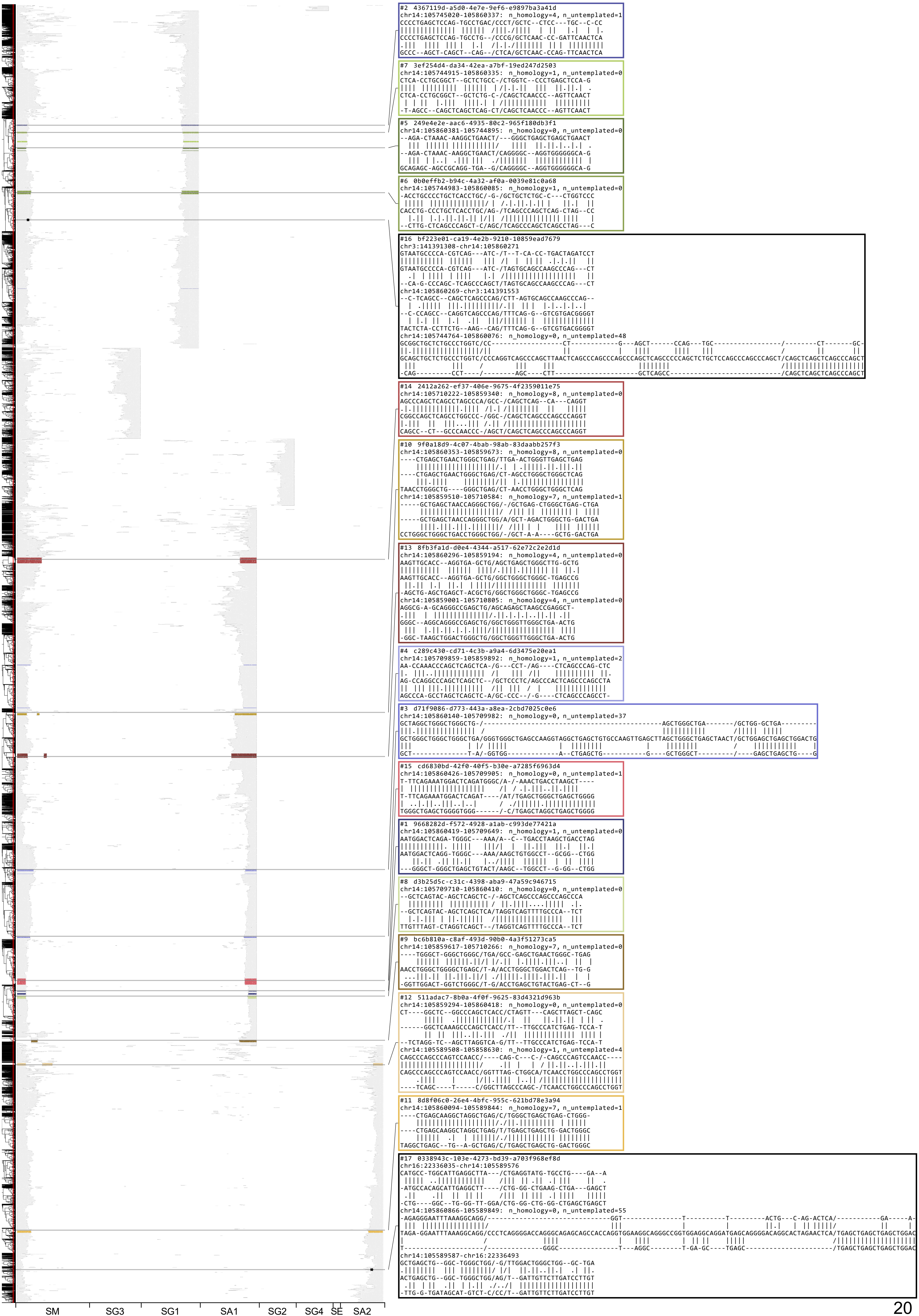

top recurring clusters in replicate 2 of 50000 B cells of donor A

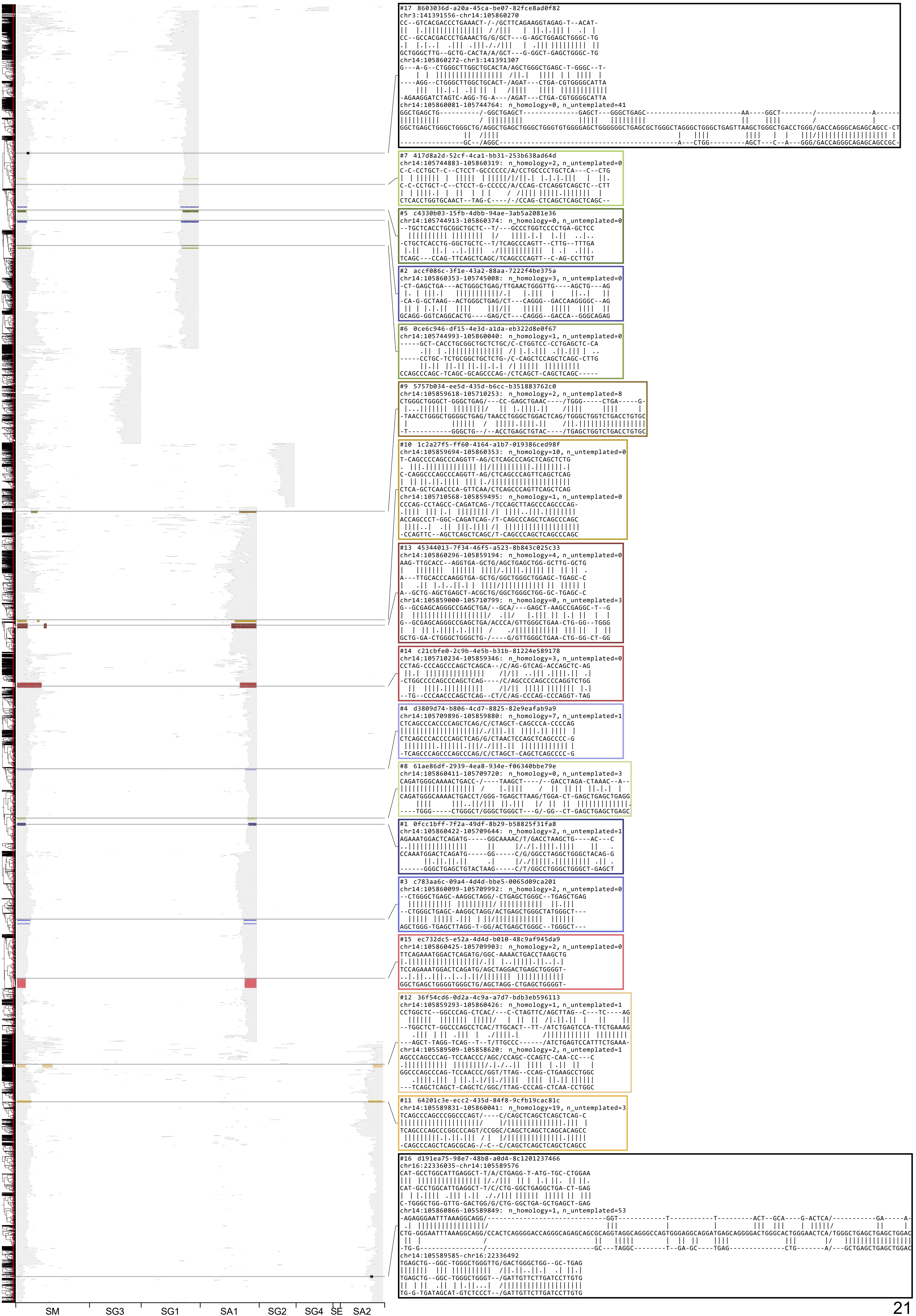

top recurring clusters in replicate 3 of 50000 B cells of donor A

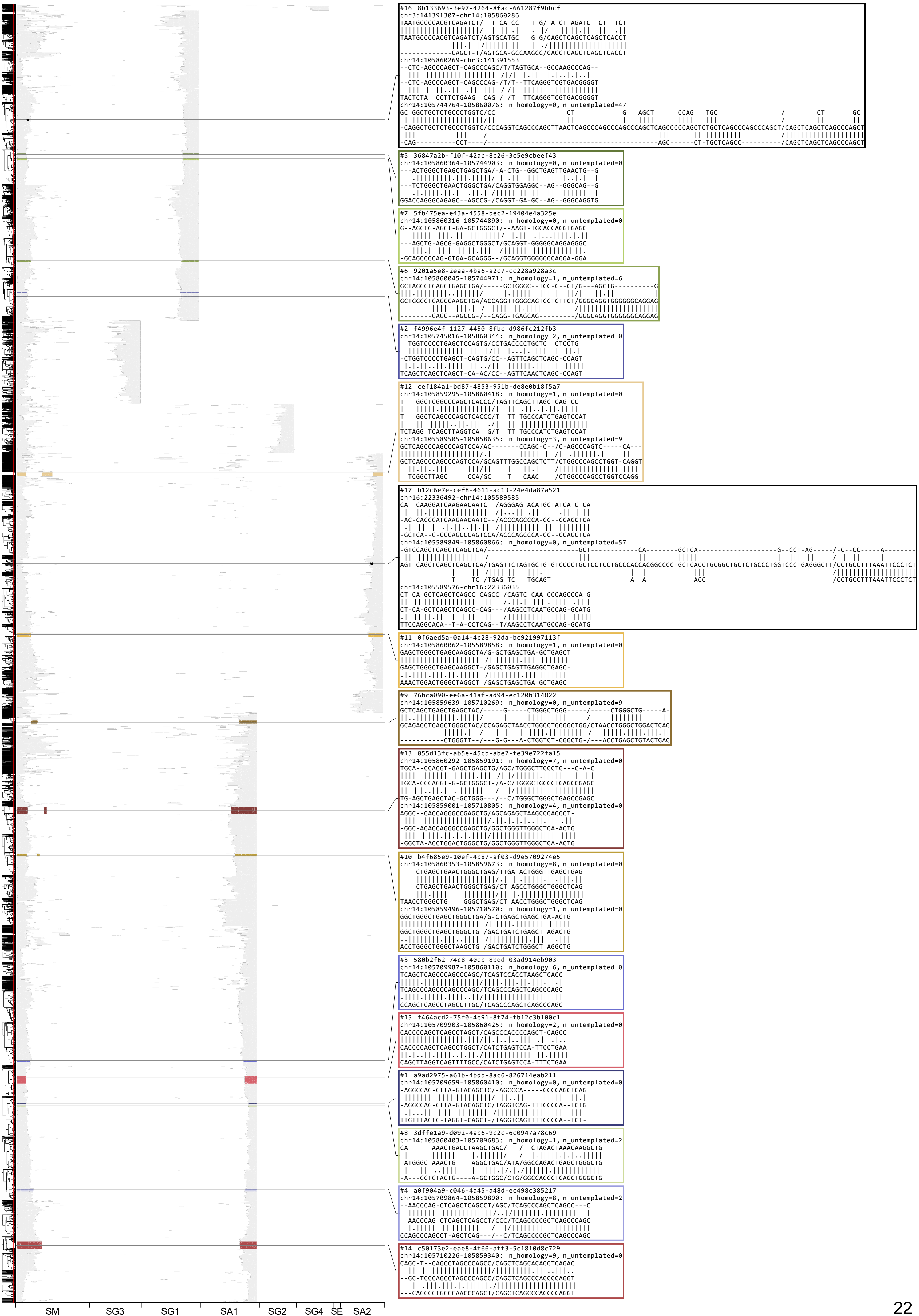

top recurring clusters for PBMCs(minION sequencing)

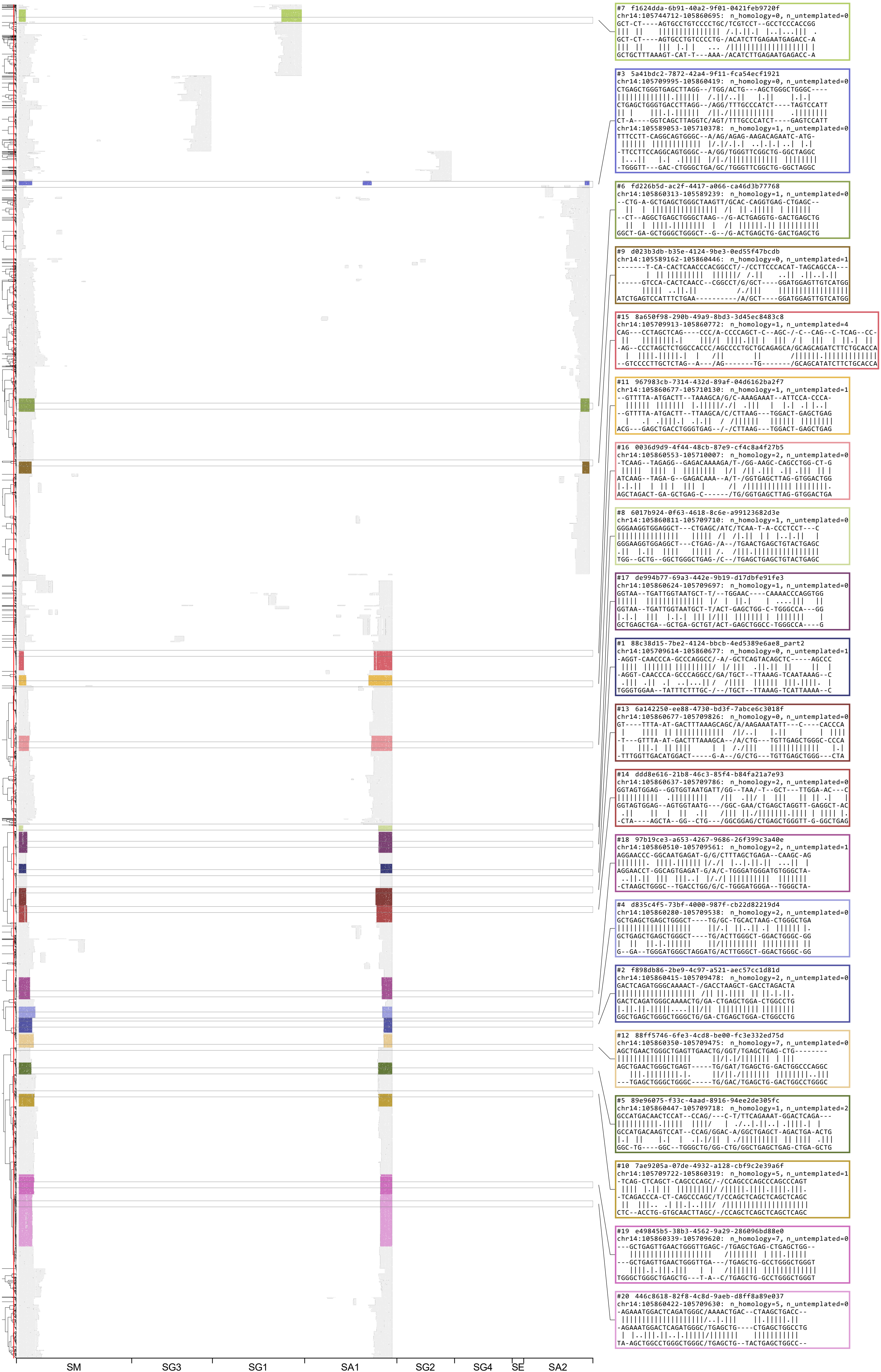

top recurring clusters for PBMCs(pacBio sequencing)

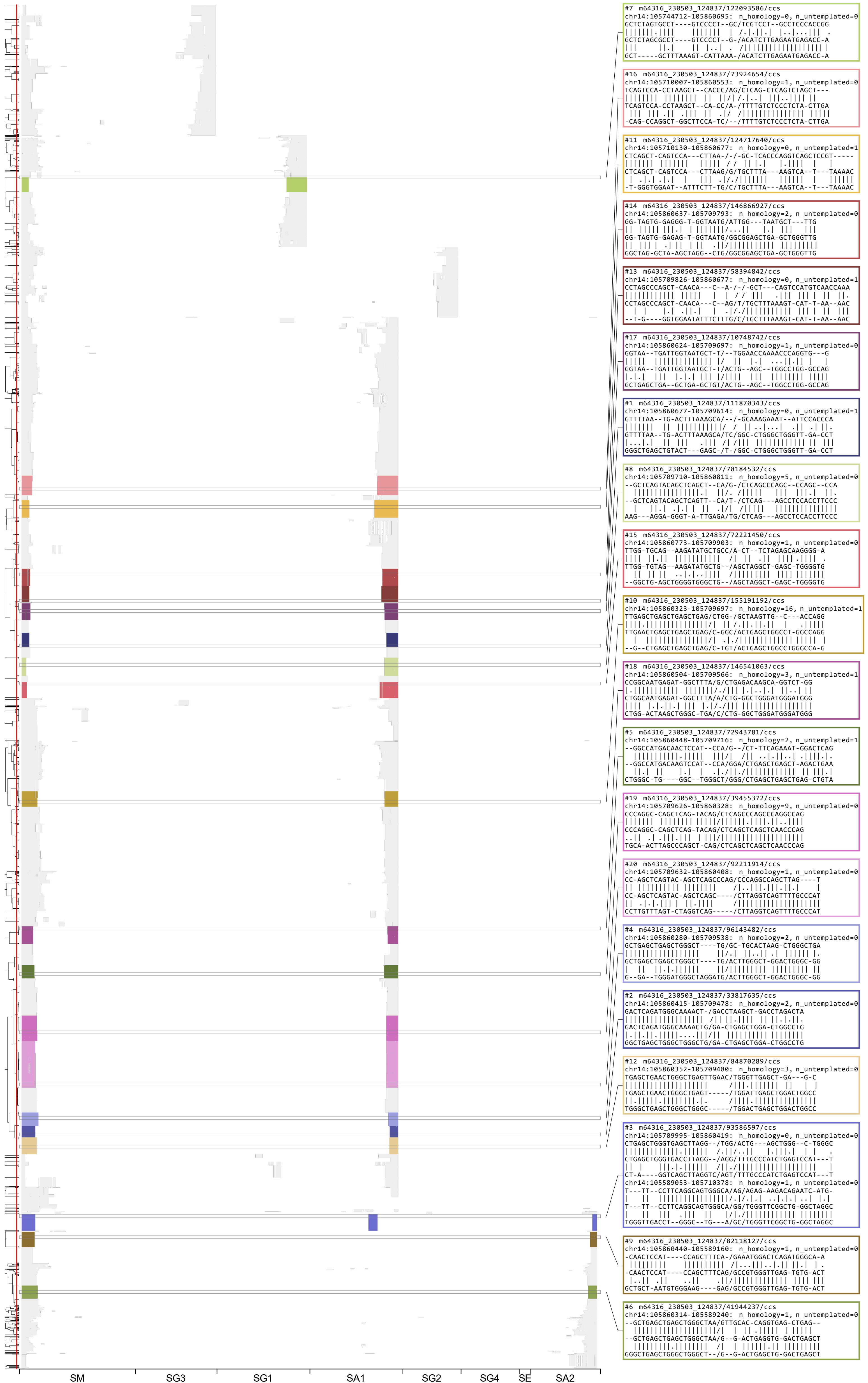

Supplementary Table 1. Primers used in CSR joint PCR

Name	Sequence	Specie	PMID
Sm FW	CACCCTTGAAAGTAGCCCATGCCTTCC	human	28847005
Sa RV	CTCAGTCCAACACCCACCACTCC	human	28847005
Sg RV	CTGCCTCCCAGTGTCCTGCATTACTTCTG	human	28847005
Se RV	GGAGGGAATGTTTTTGCAGCAGCG	human	this study
mSm FW	GGAGGGACCCAGGCTAAGAAGGC	mouse	15195091
mSa REV	GCAAGCAGTGGACCCAAAGACGAGAGG	mouse	this study
mSg2bc REV	CTGATGGGGGTGTTGTTTTGGCTG	mouse	this study
mSg3 REV	GGGCTGTTGTTGTAGCTGCAAGATAGG	mouse	this study
mSg1 REV	GCTCAGAGTGTAGAGGTCAGACTGC	mouse	this study

Supplementary Table 2. Primers used in BCR transcript sequencing

Name	Sequence	Purpose	Step
SmartNNNext	AAGCAGUGGTAUCAACGCAGAGUNNNNUNNNNUNNNNUCTTrGrGrGrG	Switch RT oligonucleotide with UMI	RT
hIGG_r1	GAAGTAGTCCTTGACCAGGCA	r1 primers Heavy Chain	
hIGM_r1	GTGATGGAGTCGGGAAGGAAG		
hIGA_r1	GCGACGACCACGTTCCCATCT		
hIGD_r1	GGACCACAGGGCTGTTATC		
hIGE_r1	AGTCACGGAGGTGGCATTG		
hIGLC_r1	GCTCCCGGGTAGAAGT	r1 primers Light Chain	
hIGKC_r1	GCGTTATCCACCTTCC		
M1ss	AAGCAGTGGTATCAACGCA	Universal primer annealing on SmartNNNext	I PCR
hIGGE_r2	ARGGGGAAGACSGATG	r2 primers Heavy Chain	
hIGA_r2	CAGCGGGAAGACCTTG		
hIGM_r2	AGGGGGAAAAGGGTTG		
hIGD_r2	ATATGATGGGGAACAC		
hIGLC_r2	GYGGGAACAGAGTGAC	r2 primers Light Chain	
hIGKC_r2	GATGGTGCAGCCACAG		
M1ss-C-U1	TCGTCGGCAGCGTCAGATGTGTATAAGAGACAGAAGCAGTGGTATCAACGCA	Universal primer with Illumina adapters	II PCR
hIGGE_r2-C-U2	GTCTCGTGGGCTCGGAGATGTGTATAAGAGACAGARGGGGAAGACSGATG	r2 primers Heavy Chain w. Illumina	
hIGA_r2-C-U2	GTCTCGTGGGCTCGGAGATGTGTATAAGAGACAGCAGCGGGAAGACCTTG		
hIGM_r2-C-U2	GTCTCGTGGGCTCGGAGATGTGTATAAGAGACAGAGGGGGAAAAGGGTTG		
hIGD_r2-C-U2	GTCTCGTGGGCTCGGAGATGTGTATAAGAGACAGATATGATGGGGAACAC		
hIGLC_r2-C-U2	GTCTCGTGGGCTCGGAGATGTGTATAAGAGACAGGYGGGAACAGAGTGAC	r2 primers Light Chain w. Illumina	
hIGKC_r2-C-U2	GTCTCGTGGGCTCGGAGATGTGTATAAGAGACAGGATGGTGCAGCCACAG		
FC-i5-index5-U1	AATGATACGGCGACCACCGAGATCTACAC XXXXXXXX TCGTCGGCAGCGTC	Illumina indexing primers	Indexing PCR
FC-i7-index7-U2	CAAGCAGAAGACGGCATACGAGAT XXXXXXXX GTCTCGTGGGCTCGG		

References:

1. Dhulipala, L., Blelloch, G. E. & Shun, J. Theoretically Efficient Parallel Graph Algorithms Can Be Fast and Scalable. arXiv (2018) doi:10.48550/arxiv.1805.05208.
