# Supplementary material for: Recombination junctions from antibody isotype switching classify immune and DNA repair dysfunction
Source: Nat Commun. 2025 Dec 19;16:11331. doi: 10.1038/s41467-025-67206-5 (PMC12722312; doi:10.1038/s41467-025-67206-5)
Supplement: Supplementary file 2 — Description of Additional Supplementary Files [file 41467_2025_67206_MOESM2_ESM.pdf]

Description of Additional Supplementary Files

Supplementary Data 1. Clonal results from tracing analysis. The three first sheets correspond to the shared clones found in replicate 1, 2 and 3 (R1, R2, R3) of donor A. The last two sheets correspond to the shared clones between two sequencing methods (minION and pacBio) from the same amplicons obtained by CSR-junction PCR.

Supplementary Data 2. CSR junction features. Description of all the features used in the SWIBRID pipeline. Information on the usage of certain features for different analysis is also indicated.

Supplementary Data 3. Description of human samples. Information on the data from the three cohorts used in Figure 4. Training samples indicate the ones used for training the machine learning (ML) model. Testing samples indicate the primary immunodeficiencies that were predicted with the ML model. Validation samples indicate the DNA repair deficient donors predicted with the ML model.
