# Supplementary material for: Recombination junctions from antibody isotype switching classify immune and DNA repair dysfunction
Source: Nat Commun. 2025 Dec 19;16:11331. doi: 10.1038/s41467-025-67206-5 (PMC12722312; doi:10.1038/s41467-025-67206-5)
Supplement: Supplementary file 6 — Reporting Summary [file 41467_2025_67206_MOESM6_ESM.pdf]

nature portfolio

Corresponding author(s): Kathrin de la Rosa

Last updated by author(s): 13.11.2025

Reporting Summary

Nature Portfolio wishes to improve the reproducibility of the work that we publish. This form provides structure for consistency and transparency in reporting. For further information on Nature Portfolio policies, see our Editorial Policies and the Editorial Policy Checklist.

Statistics

For all statistical analyses, confirm that the following items are present in the figure legend, table legend, main text, or Methods section.

n/a	Confirmed	
☐	☒	The exact sample size (*n*) for each experimental group/condition, given as a discrete number and unit of measurement
☐	☒	A statement on whether measurements were taken from distinct samples or whether the same sample was measured repeatedly
☐	☒	The statistical test(s) used AND whether they are one- or two-sided<br>*Only common tests should be described solely by name; describe more complex techniques in the Methods section.*
☐	☒	A description of all covariates tested
☐	☒	A description of any assumptions or corrections, such as tests of normality and adjustment for multiple comparisons
☐	☒	A full description of the statistical parameters including central tendency (e.g. means) or other basic estimates (e.g. regression coefficient) AND variation (e.g. standard deviation) or associated estimates of uncertainty (e.g. confidence intervals)
☐	☒	For null hypothesis testing, the test statistic (e.g. F, t, r) with confidence intervals, effect sizes, degrees of freedom and P value noted<br>*Give P values as exact values whenever suitable.*
☒	☐	For Bayesian analysis, information on the choice of priors and Markov chain Monte Carlo settings
☒	☐	For hierarchical and complex designs, identification of the appropriate level for tests and full reporting of outcomes
☐	☒	Estimates of effect sizes (e.g. Cohen's *d*, Pearson's *r*), indicating how they were calculated

Our web collection on statistics for biologists contains articles on many of the points above.

Software and code

Policy information about availability of computer code

Data collection	SWIBRID code and documentation is available at github.com/bihealth/swibrid (http://dx.doi.org/10.5281/zenodo.17415052) and bihealth.github.io/swibrid. Scripts used to analyze data in this manuscript are available at github.com/bihealth/swibrid_paper (http://dx.doi.org/10.5281/zenodo.17415060). Used software and packages include BLAST (v2.14), fastcluster (v1.2.6), LAST (v1453), numpy (v1.24.3), pandas (v2.0.1), pysam (v0.22.0), python (v3.8.16), scikit-learn (v1.2.0), scipy (v1.9.3), snakemake (v7.8.1), wNMF (v0.0.42), bwa-mem2 (v2.2.1).
Data analysis	Data analysis conducted in R (v4.3.2) using packages variancePartition (v1.32.5), pROC (v1.18.5), glmnet (v4.1-8), ggrepel (v0.9.5), ComplexHeatmap (v2.18.0), caret (v6.0-94), tidyr (v1.3.1), tidyverse (v2.0.0), dplyr (v1.1.4), ggpubr (v0.6.0), ggplot2 (v3.5.1), ggtools (v3.9.5) and dendextend (v1.17.1).

For manuscripts utilizing custom algorithms or software that are central to the research but not yet described in published literature, software must be made available to editors and reviewers. We strongly encourage code deposition in a community repository (e.g. GitHub). See the Nature Portfolio guidelines for submitting code & software for further information.

Data

Policy information about availability of data

All manuscripts must include a data availability statement. This statement should provide the following information, where applicable:

- Accession codes, unique identifiers, or web links for publicly available datasets
- A description of any restrictions on data availability
- For clinical datasets or third party data, please ensure that the statement adheres to our policy

Raw sequencing data from mouse samples are available at SRA (accession number PRJNA1190672). Raw data for human samples cannot be shared publicly in accordance with patient privacy legislation. SWIBRID output for these samples, anonymized as required by the respective Ethics statement, is available at github.com/bihealth/swibrid_paper (http://dx.doi.org/10.5281/zenodo.17415060)

Research involving human participants, their data, or biological material

Policy information about studies with human participants or human data. See also policy information about sex, gender (identity/presentation), and sexual orientation and race, ethnicity and racism.

Reporting on sex and gender	Sex was self-reported by study participants. Its relevance was tested using variance decomposition and subsequently neglected from further analysis.
Reporting on race, ethnicity, or other socially relevant groupings	No information regarding the race or ethnicity was obtained from the patient and control samples obtained by the German Red Cross.
Population characteristics	Age, sex, diagnoses and genotype status of study participants where applicable are reported in Supplementary Table 2.
Recruitment	The obtainment of control samples from the German Red Cross was random and did not involve any special recruitment.
Ethics oversight	Studies were approved by the Charité Ethical Commission (EA1/149/23), the local authorities of Freiburg, Germany (251/13 & 254/19), the Institutional Review Board of Karolinska Institutet and the Ethics Committee of the Tehran University of Medical Sciences (IR.TUMS.CHMC.REC.1398.030).

Note that full information on the approval of the study protocol must also be provided in the manuscript.

Field-specific reporting

Please select the one below that is the best fit for your research. If you are not sure, read the appropriate sections before making your selection.

☒ Life sciences ☐ Behavioural & social sciences ☐ Ecological, evolutionary & environmental sciences

For a reference copy of the document with all sections, see nature.com/documents/nr-reporting-summary-flat.pdf

Life sciences study design

All studies must disclose on these points even when the disclosure is negative.

Sample size	No sample size calculation was performed. For Figure 3, we considered that a total of 49 samples (at least 9 per genotype) represent every sample with enough characteristics, since CH12 is a cell line. In Figure 4, human samples were subjected to availability by our collaborators. We made sure that the samples for the training dataset could have 60% heterogenous patients and 40% controls. Datasets to predict also contained similar number of patients and control samples.
Data exclusions	Samples with less than 500 (post-filter) reads or those failing visual quality control using the read plots were excluded.
Replication	Machine learning experiments were performed using separate: training (n=35) and testing (n=26 & n=21) datasets to confirm cross-validation. There were two testing datasets, one coming from the same cohort, from where the training dataset came, and the other from a complete different one. The latter contained mostly DNA repair deficiencies, which also trigger immunodeficiencies in the majority of the cases, while the other datasets were comprised of immunodeficiencies. The machine learning trained with immunodeficient samples, was able to identify the immunodeficient separate dataset with a 99% accuracy and the DNA repair deficient dataset with a 84% accuracy, confirming that the machine learning can reproduce the identification of disease versus control.
Randomization	Control cohorts were collected aiming for comparable representation of male/female and older/younger donors. Samples from training and testing datasets from Figure 4, were not randomly selected. They were selected considering the patient consent and the material availability.
Blinding	Parts of the dataset used in Figure 3 and 4 were provided without genotype or diagnosis meta-data for initial stages of data analysis. Specifically, the scientist performing the laboratory and computational analysis were blinded when analyzing samples from the testing and DNA repair deficient donors (Figure 4e, f). The scientist performing the computational analysis was blinded when analyzing the data from the samples in Figure 3g, Figure 4d, g.

Reporting for specific materials, systems and methods

We require information from authors about some types of materials, experimental systems and methods used in many studies. Here, indicate whether each material, system or method listed is relevant to your study. If you are not sure if a list item applies to your research, read the appropriate section before selecting a response.

Materials & experimental systems

n/a	Involved in the study
☐	☒ Antibodies
☐	☒ Eukaryotic cell lines
☒	☐ Palaeontology and archaeology
☐	☒ Animals and other organisms
☐	☒ Clinical data
☒	☐ Dual use research of concern
☒	☐ Plants

Methods

n/a	Involved in the study
☒	☐ ChIP-seq
☒	☐ Flow cytometry
☒	☐ MRI-based neuroimaging

Antibodies

Antibodies used

These antibodies were used in flow cytometry in human lymphocytes: CD27-PE (Miltenyi Biotec, #130-114-156, clone M-T271); IgD-PE-Cy7(Miltenyi Biotec, #130-098-584), IgM-AF488(Life Technologies, #A21215); IgG-AF647(Dianova, #109-606-170); IgA-AF647 (Dianova, #109-606-01); IgA-APC-Vio770 (Miltenyi Biotec, #130-113-473, clone IS11-8), CD19-Brilliant Violet 6005 (Becton Dickinson, #562653); CD20-PerCP-Vio700 (Miltenyi Biotec, #130-113-377, clone LT20).
This antibody was used to activate CH12 cells: anti-mouse CD40 (BIOZOL, #BLD-102902)

Validation

References of validation can be found in the manufacturer´s website:

- CD27-PE (Miltenyi Biotec, #130-114-156) - www.miltenyibiotec.com/DE-en/products/cd27-antibody-anti-human-m-t271.html#conjugate=viogreen:size=100-tests-in-200-ul
- IgD-PE-Cy7(Miltenyi Biotec, #130-098-584) - www.miltenyibiotec.com/DE-en/products/igd-antibody-anti-human-igd26.html#conjugate=fitc:size=100-tests-in-200-ul
- IgM-AF488(Life Technologies, #A21215) - www.thermofisher.com/antibody/product/Goat-anti-Human-IgM-Heavy-chain-Cross-Adsorbed-Secondary-Antibody-Polyclonal/A-21215
- IgG-AF647(Dianova, #109-606-170) - www.dianova.com/shop/109-606-170-ziege-fab2-anti-human-igg-fc-alexa-fluor-647-minx-bomsrb/
- IgA-AF647 (Dianova, #109-606-01) - www.dianova.com/shop/109-606-011-ziege-fab2-anti-human-iga-alexa-fluor-647-minx-keine/?_gl=1*a4p647*_up*MQ..*_ga*MTk1ODU1ODc5My4xNzYxMzA5NTUz*_ga_MVW3JQ2V5D*czE3NjEzMDk1NTlkbzEkZzEkdDE3NjEzMDk1OTEkajIxJGwwJGgw
-IgA-APC-Vio770 (Miltenyi Biotec, #130-113-473, clone IS11-8) - https://www.miltenyibiotec.com/DE-en/products/iga-antibody-anti-human-is11-8e10.html#conjugate=viogreen:size=100-tests-in-200-ul
- CD19-Brilliant Violet 605 (Becton Dickinson, #562653) - https://www.bdbiosciences.com/en-de/products/reagents/flow-cytometry-reagents/research-reagents/single-color-antibodies-ruo/bv605-mouse-anti-human-cd19.562653?tab=product_details
-CD20-PerCP-Vio700 (Miltenyi Biotec, #130-113-377) - https://www.miltenyibiotec.com/US-en/products/cd20-antibody-anti-human-lt20.html#conjugate=viogreen:size=100-tests-in-200-ul
- anti-mouse CD40 (BIOZOL, #BLD-102902) - www.biolegend.com/en-gb/products/purified-anti-mouse-cd40-antibody-290?GroupID=BLG11938

Eukaryotic cell lines

Policy information about cell lines and Sex and Gender in Research

Cell line source(s)

CH12 F3 cell line was obtained from the laboratory of Prof. Dr. Michela Di Virgilio

Authentication

CH12 F3 are the only B cells that are immortalized and can perform class-switch recombination. Therefore, cells were activated using IL-4, anti-CD40 antibody and TGFB1 cocktail with successful IgA class switching via flow cytometry and switch-PCR.

Mycoplasma contamination

Supernatant of culture was checked and no mycoplasma was found.

Commonly misidentified lines
(See ICLAC register)

No commonly misidentified cell lines were used in this study.

Animals and other research organisms

Policy information about studies involving animals; ARRIVE guidelines recommended for reporting animal research, and Sex and Gender in Research

Laboratory animals	C57BL/6 mice aged 9 to 10 weeks.
Wild animals	No wild animals were used in this study.
Reporting on sex	All mice were female.
Field-collected samples	No field-collected samples were used in this study.
Ethics oversight	Animal experiments were carried out in accordance with the of the German Animal Protection Law and approved by the local responsible authorities. Experimental Pharmacology & Oncology Berlin-Buch GmbH (EPO) (Ther. E0023/23, study number: 18906) performed the animal experiments. EPO complies to the EU guideline “European convention for the protection of vertebrate animals used for experimental and other scientific purposes. (EST 123)”. The female C57BL/6 mice were handled in EPO GmbH according to the “Regulation on the protection of experimental scientific purposes or other Purposes used animals”. Compliance with the above rules and regulations is monitored by the Landesamt fuer Gesundheit und Soziales (LAGeSo) which is the responsible regulatory authority monitoring the animal husbandry based on the German Animal Welfare Act. Approval was given after careful inspection of the site including bedding, feeding & water, ventilation, temperature, and humidity, cleaning and hygiene concepts.

Note that full information on the approval of the study protocol must also be provided in the manuscript.

Clinical data

Policy information about clinical studies

All manuscripts should comply with the ICMJE guidelines for publication of clinical research and a completed CONSORT checklist must be included with all submissions.

Clinical trial registration	*Provide the trial registration number from ClinicalTrials.gov or an equivalent agency.*
Study protocol	*Note where the full trial protocol can be accessed OR if not available, explain why.*
Data collection	*Describe the settings and locales of data collection, noting the time periods of recruitment and data collection.*
Outcomes	*Describe how you pre-defined primary and secondary outcome measures and how you assessed these measures.*

Plants

Seed stocks	*Report on the source of all seed stocks or other plant material used. If applicable, state the seed stock centre and catalogue number. If plant specimens were collected from the field, describe the collection location, date and sampling procedures.*
Novel plant genotypes	*Describe the methods by which all novel plant genotypes were produced. This includes those generated by transgenic approaches, gene editing, chemical/radiation-based mutagenesis and hybridization. For transgenic lines, describe the transformation method, the number of independent lines analyzed and the generation upon which experiments were performed. For gene-edited lines, describe the editor used, the endogenous sequence targeted for editing, the targeting guide RNA sequence (if applicable) and how the editor was applied.*
Authentication	*Describe any authentication procedures for each seed stock used or novel genotype generated. Describe any experiments used to assess the effect of a mutation and, where applicable, how potential secondary effects (e.g. second site T-DNA insertions, mosiacism, off-target gene editing) were examined.*
